# Serodominant SARS-CoV-2 Nucleocapsid Peptides Map to Unstructured Protein Regions

**DOI:** 10.1128/spectrum.00324-23

**Published:** 2023-05-16

**Authors:** Jacob P. Vandervaart, Nicole L. Inniss, Ted Ling-Hu, George Minasov, Grant Wiersum, Monica Rosas-Lemus, Ludmilla Shuvalova, Chad J. Achenbach, Judd F. Hultquist, Karla J. F. Satchell, Kelly E. R. Bachta

**Affiliations:** a Department of Microbiology-Immunology, Northwestern University, Feinberg School of Medicine, Chicago, Illinois, USA; b Robert H. Lurie Comprehensive Cancer Center, Northwestern University, Feinberg School of Medicine, Chicago, Illinois, USA; c Center for Structural Biology of Infectious Diseases, Northwestern University, Feinberg School of Medicine, Chicago, Illinois, USA; d Department of Medicine, Division of Infectious Diseases, Northwestern University, Feinberg School of Medicine, Chicago, Illinois, USA; e Department of Pharmacology, Northwestern University, Feinberg School of Medicine, Chicago, Illinois, USA; University of Arizona

**Keywords:** SARS-CoV-2, nucleocapsid, three-dimensional structure, immune response, epitope, variants of concern

## Abstract

The SARS-CoV-2 nucleocapsid (N) protein is highly immunogenic, and anti-N antibodies are commonly used as markers for prior infection. While several studies have examined or predicted the antigenic regions of N, these have lacked consensus and structural context. Using COVID-19 patient sera to probe an overlapping peptide array, we identified six public and four private epitope regions across N, some of which are unique to this study. We further report the first deposited X-ray structure of the stable dimerization domain at 2.05 Å as similar to all other reported structures. Structural mapping revealed that most epitopes are derived from surface-exposed loops on the stable domains or from the unstructured linker regions. An antibody response to an epitope in the stable RNA binding domain was found more frequently in sera from patients requiring intensive care. Since emerging amino acid variations in N map to immunogenic peptides, N protein variation could impact detection of seroconversion for variants of concern.

**IMPORTANCE** As SARS-CoV-2 continues to evolve, a structural and genetic understanding of key viral epitopes will be essential to the development of next-generation diagnostics and vaccines. This study uses structural biology and epitope mapping to define the antigenic regions of the viral nucleocapsid protein in sera from a cohort of COVID-19 patients with diverse clinical outcomes. These results are interpreted in the context of prior structural and epitope mapping studies as well as in the context of emergent viral variants. This report serves as a resource for synthesizing the current state of the field toward improving strategies for future diagnostic and therapeutic design.

## INTRODUCTION

The ongoing coronavirus disease 2019 (COVID-19) pandemic has resulted in over 6.7 million deaths globally, with more than 1 million in the United States alone (https://coronavirus.jhu.edu). This severe respiratory disease is caused by severe acute respiratory syndrome coronavirus 2 (SARS-CoV-2), a novel coronavirus that emerged in late 2019 (1). As of early 2022, large-scale seroprevalence studies have estimated that more than 60% of Americans have been infected (2). These studies are largely based on serological testing for antibodies against the SARS-CoV-2 nucleocapsid (N) protein. This viral protein is highly abundant during acute infection and highly immunogenic, resulting in a strong and relatively ubiquitous antibody response, even in asymptomatic or mild cases (2–5). Critically, the N protein is not present in the COVID-19 vaccines authorized for use in the United States, and thus, the detection of anti-N antibodies is specifically indicative of prior infection (2).

**FIG 1:**
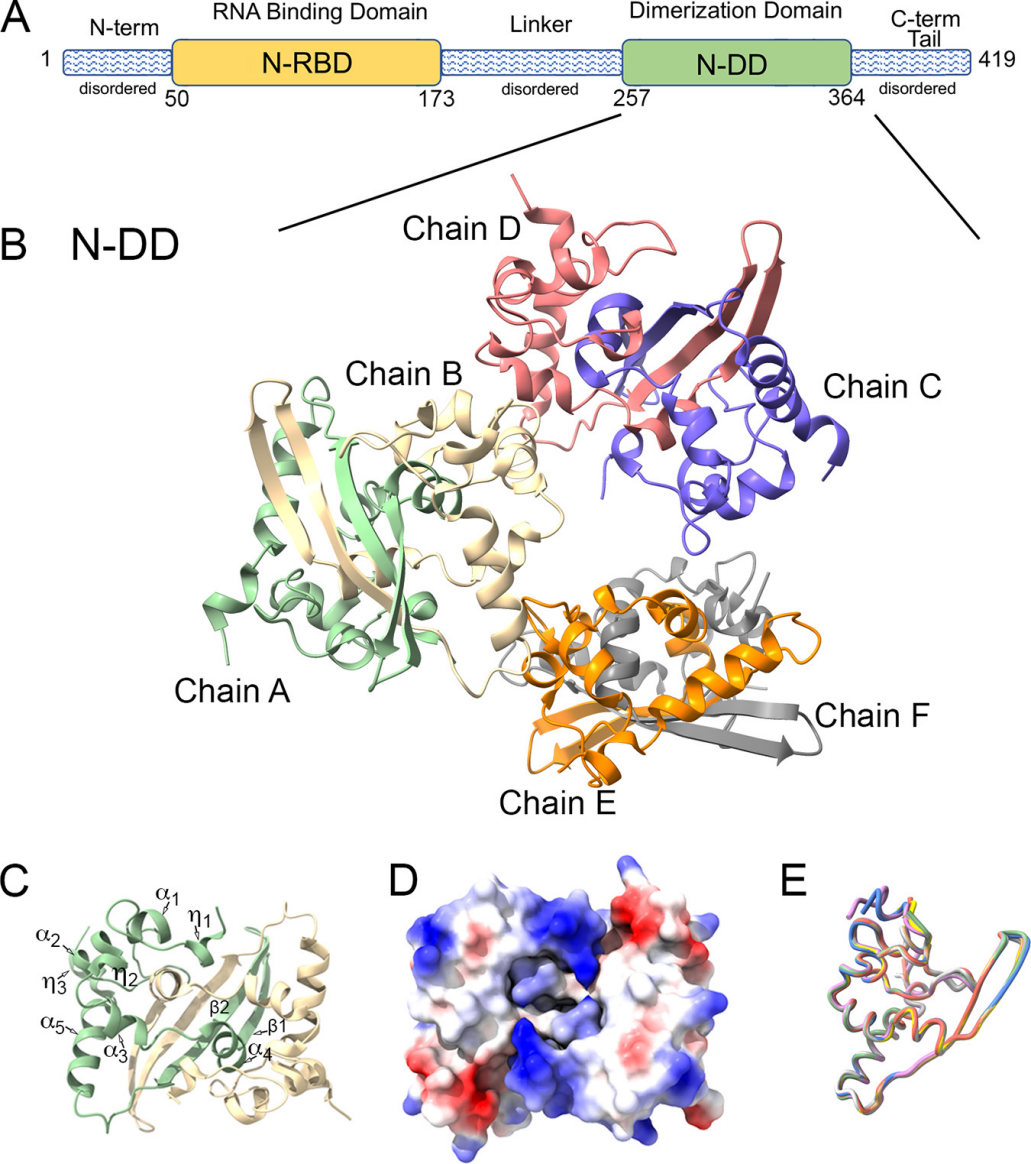
X-ray structure of N protein C-terminal dimerization domain. (A) Schematic diagram of the N protein with distinct domains. (B) X-ray structure of N protein N-DD solved at 2.05 Å shown as a cartoon with three dimers in the asymmetric unit (PDB accession no. 6WJI). (C) Chains A (green) and B (beige) shown as a dimer. Chain A is labeled with secondary structure elements. (D) Electrostatic projection of dimer in the same orientation as panel C. (E) Overlay licorice diagram of structure PDB accession no. 6WJI chain A (green) with other N-DD protein structures, PDB accession no. 6WZQ (blue), 6WZO (tan), 6YUN (violet), 7CE0 (yellow), 7C22 (orange), and 7DE1 (gray)

The SARS-CoV-2 N protein binds to viral genomic RNA and oligomerizes around it to form a closed capsule that both protects the genome from antiviral responses and directs its packaging into new virions (6, 7). Beyond its role in nucleocapsid assembly and packaging, the N protein is also important during viral RNA synthesis, where it binds double-stranded RNA during viral genome replication and participates in the discontinuous transcription process necessary to generate subgenomic mRNAs (8, 9). The protein is comprised of five domains (Fig. 1A). There are two stable, proteolysis-resistant domains, the N-terminal RNA binding domain (N-RBD), which binds to virus genomic RNA, and the dimerization domain (N-DD), which facilitates protein oligomerization and has some nonspecific RNA binding activity. These two domains are interspersed with intrinsically disordered domains designated the N-terminal arm (NTD), linker region (linker), and C-terminal tail (Fig. 1A) (10–13).

Antibodies targeting the N protein or the spike (S) protein can be detected in as few as 1 to 2 weeks following symptom onset and generally remain detectable up to a year after infection, with estimated seroconversion rates of roughly 50% after 2 years (14), though this varies widely with detection method (15). Several studies have reported that higher anti-SARS-CoV-2 antibody titers are associated with more severe outcomes (16–18), though this may be dependent on time since symptom onset, immunoglobin, and antigen (17, 19). A few studies have reported that higher anti-N antibody responses are associated with more severe outcomes, particularly with hospitalizations of long duration (20–22). While several reports have mapped or predicted antigenic regions within the N protein, these often lack consensus, and only a handful have compared antigenic regions by patient outcome (23–32).

In April 2020, we reported the first crystal structure of the N protein N-DD from SARS-CoV-2, which was made immediately accessible through the RCSB Protein Data Bank, along with structures solved by others of the same protein domain (Table 1). In the manuscript, we report the methods and statistics of this crystal structure that has been widely used in efforts to understand N protein structure and function. Furthermore, with sera collected from 34 COVID-19 patients between March and April of 2020 during the first wave of the pandemic; we mapped highly prevalent, serodominant antigenic epitopes in the N protein using a peptide-based enzyme-linked immunosorbent assay (ELISA) with 10-amino-acid resolution. These data were used to identify patient groups who produced distinct antibody responses to various peptide signatures within the N protein. Combined analysis of these peptide signatures with the structure revealed that all immunodominant peptides were derived from unstructured regions of the protein or surface-localized loops of the stable domains, including an epitope in N-DD not recognized by prior studies. These data are contextualized by a literature review of N protein epitope mapping and amino acid (aa) variation efforts to date summarizing the current state of the field.

**TABLE 1:** All publicly available X-ray structures of N protein-stable domains^*a*^

Domain	PDB accession no.	Resolution (Å)	pH	Public release date (yr-mo-day)	Reference or source
N-RBD	6VYO	1.7	6.0	2020-03-11	Unpublished
	6M3M	2.7	6.5	2020-03-18	49
	6WKP	2.67	6.5	2020-04-29	Unpublished
	7CDZ	1.8		2020-09-02	50
	7VNU	1.95	7.5	2021-10-27	Unpublished
	7VBD	1.94	8.0	2022-08-31	Unpublished
N-DD	6WJI	2.05	7.5	2020-04-22	This study
	7C22	2.0		2020-05-20	51
	6YUN	1.44	7.8	2020-05-20	33
	6WZO	1.42		2020-05-27	52
	6WZQ	1.45	8.5	2020-05-27	52
	6ZCO	1.361		2020-07-01	33
	7CE0	1.5	9.0	2020-09-02	50
	7DE1	2.0	9.3	2021-01-27	53
	7F2B	2.0	6.2	2021-09-01	54
	7F2E	3.1	4.5	2021-10-20	54
	7O05	1.94		2022-04-13	55
	7UXX	1.85	8.3	2022-06-22	56
	7VBE	1.59	5.0	2022-07-06	Unpublished
	7VBF	1.3	8.5	2022-07-06	Unpublished

a Excluding proteins in complex with antibodies or ligands.

## RESULTS

### Description of the first publicly released X-ray crystal structure for SARS-CoV-2 N-DD.

Since the start of the COVID-19 pandemic, there has been a worldwide effort to structurally characterize each of the SARS-CoV-2 viral proteins. While the structure of the full-length N protein is yet to be fully resolved, several structures of each of the stable, proteolysis-resistant domains have been solved (Table 1). We determined the first publicly available structure of SARS-CoV-2 N-DD (residues 247 to 364), which was released on 22 April 2020 to the Protein Data Bank with accession no. 6WJI (Table 1). The crystal belonged to the *P*2_1_2_1_2_1_ space group (cell parameters, *a = *43.61, *b = *122.32, *c *= 130.63) and was refined to 2.05 Å resolution. The asymmetric unit consisted of six polypeptide chains, which formed three homodimers (Fig. 1B). Each protomer contained 108 residues (257 to 364) that formed 5 α helices, α_1_ (269 to 275), α_2_ (288 to 295), α_3_ (301 to 306), α_4_ (310 to 318), and α_5_ (345 to 357); 3 η helices, η_1_ (258 to 262), η_2_ (296 to 298), and η_3_ (358 to 363); and 2 β-strands, β_1_ (319 to 325) and β_2_ (328 to 338), all of which were connected by five loops (Fig. 1C).

The SARS-CoV-2 N-DD homodimer resembles a rectangular prism (Fig. 1C) with a buried surface area of ~5,274 Å^2^. One large interface is formed by a four-stranded, antiparallel β-sheet, comprised of the paired protomer β-hairpins (β_1_-β_2_–β′_1_-β′_2_) (best depicted between chains A and B [Fig. 1B]). A second large interface is formed by interlocked helices and loops (α_4_-η′_1_) (best depicted between chains C and D [Fig. 1B]). The structure has nine chloride ions bound (see Table S1 in the supplemental material), which were positioned on the positively charged helical face of each dimer (Fig. 1D).

The overall topology observed in our structure was essentially identical to the 13 subsequently deposited structures of the SARS-CoV-2 N-DD (Table 1). The structural alignment of our structure with other representative structures showed that the backbones were nearly identical (Fig. 1E). Our structure was most similar to the high-resolution structure (PDB accession no. 6YUN) solved by Zinzula et al. (33), with a root mean square deviation (RMSD) of 0.230 Å across all 108 pairs.

### Identification of public epitopes in patient sera by tiled peptide ELISAs.

The relatively high abundance and immunogenicity of the SARS-CoV-2 N protein make it a major antigen presented during acute infection (3, 24). To better understand the antigenic regions of the protein, we used an ELISA-based assay to test for the presence of serum antibodies against a tiled array of 59 overlapping linear peptides spanning the N protein (Table S2). Each array was independently probed in duplicate with serum collected from a total of 34 individuals hospitalized with COVID-19 between March and April of 2020 (cohort previously reported by Simons et al. [34]). Specimens from early in the COVID-19 pandemic were chosen for three reasons, (i) these early specimens precluded potential confounding effects due to vaccination or reinfection, (ii) circulating clades at this time had limited diversity, which minimized confounding effects due to mismatches between the peptide array and the patient SARS-CoV-2 isolate, and (iii) these specimens were previously shown to harbor antibodies against the N protein. Study participants included 20 patients admitted to the intensive care unit (ICU) and 14 patients who were admitted to the hospital but never admitted to the ICU. Serum was collected in a range from 3 to 44 days following symptom onset (median, 12.5 days; interquartile range, 9 to 16.75 days). Sera collected from three healthy individuals prior to the pandemic served as negative controls alongside a blank “no serum” control.

**FIG 2:**
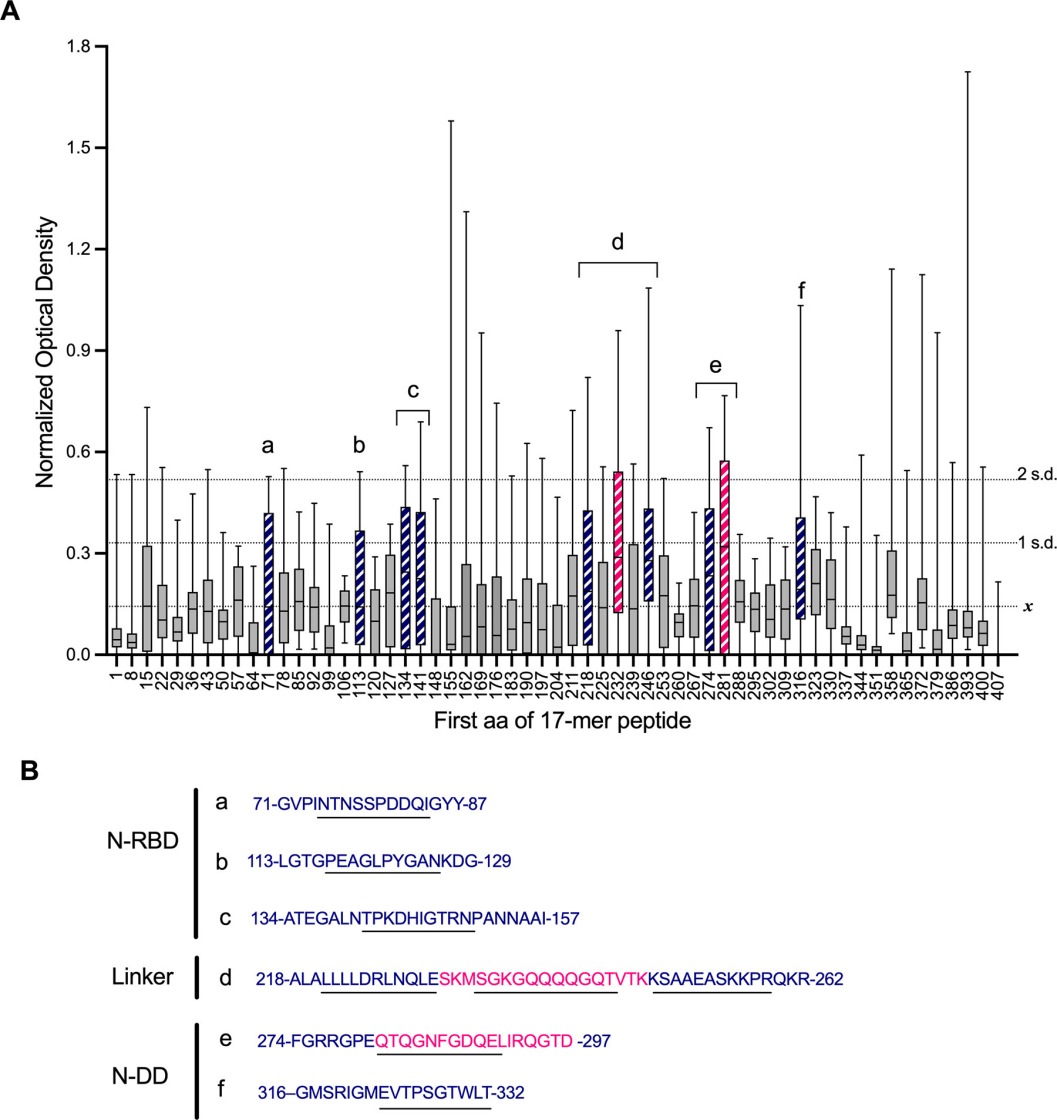
Public epitopes in the SARS-CoV-2 N protein. (A) Normalized serum ELISA data (optical density) from 34 patients shown as a whisker plot from minimum to maximum, with boxes representing the 25% and 75% quartiles with the bar set at the median. Magenta hatched bars indicate that the median optical density was at least 1 standard deviation (1 SD) above the mean (*x*) of all data, and the upper quartile exceeded 2 SDs above the mean. Blue bars indicate that the median was above *x*, and the upper quartile exceeded 1 SD above the mean. (B) Sequences of antigenic peptides are listed with peptides resulting in a modest response in blue and a strong response in magenta. Overlapping 10- or 11-mer sequences are underlined. Regions a to f are labeled in order of sequence of N.

Previous reports have shown that several immunodominant regions of the N protein result in an antibody response in a majority of COVID-19 patients (deemed “public” epitopes), while other regions elicit a response in only a few individuals (deemed “private” epitopes) (24). To identify public epitopes detectable in participant sera, the normalized absorbance across the 34 serum samples for each peptide was plotted and interpreted relative to the mean and standard deviation of all absorbance data in the data set (see Materials and Methods). Peptides with the upper quartile of the normalized absorbance greater than two standard deviations above the mean of all data were considered highly prevalent and highly reactive (Fig. 2A, magenta bars; *n *= 2 peptides). Peptides with an upper quartile greater than one standard deviation above the mean of all data were considered highly prevalent and moderately reactive (Fig. 2A, blue bars; *n *= 8 peptides). All 10 peptides were deemed public epitopes.

Two peptides in the N protein were highly prevalent and highly reactive, peptide 34 (aa 232 to 248, SKMSGKGQQQQGQTVTK) and peptide 41 (aa 281 to 297, QTQGNFGDQELIRQGTD) (Fig. 2B, magenta sequences). Peptide 34 bridges the end of the linker domain and the beginning of the N-DD. The neighboring peptides to either side of peptide 34 (peptides 33 and 35) were less reactive, indicating that the most antigenic sequence within this region was contained solely within peptide 34 in the nonoverlapping sequence (Fig. 2B, underlined, aa 235 to 245, SGKGQQQQGQT). However, two highly prevalent and moderately reactive peptides, peptides 32 (aa 218 to 234) and 36 (aa 246 to 262), are closely adjacent, suggesting the entire region may be antigenic (Fig. 2A, region d). The other highly prevalent, highly antigenic peptide, peptide 41 (aa 281 to 297, QTQGNFGDQELIRQGTD), was also located in the N-DD. The neighboring peptides (peptides 42 and 43, aa 288 to 311) to the C-terminal side did not elicit a strong response, though moderately reactive peptide 40 (aa 274 to 290) overlaps the N-terminal side (Fig. 2A and B, region e). This suggests that the most antigenic sequence may be in the 10-aa overlap between the peptides (Fig. 2B, underlined, aa 281 to 290, QTQGNFGDQE).

Five additional highly prevalent and moderately reactive public epitopes were also identified, including peptides 11 (aa 71 to 87, region a), 17 (aa 113 to 129, region b), 20 (aa 134 to 150, region c), and 21 (aa 141 to 157, also region c) in the N-RBD, as well as peptide 46 (aa 316 to 332, region f) in the N-DD (Fig. 2A and B).

### Clustering of patient antibody profiles identified three additional private immunogenic regions.

In addition to the public epitopes, there were also several instances of peptides that induced strong antibody reactions, but in fewer patient samples. To better characterize these highly reactive but less prevalent “private” epitopes, we used principal-component analysis (PCA) and *k*-means clustering to identify groups of participants with similar antibody responses and visualized these responses in a heatmap.

**FIG 3:**
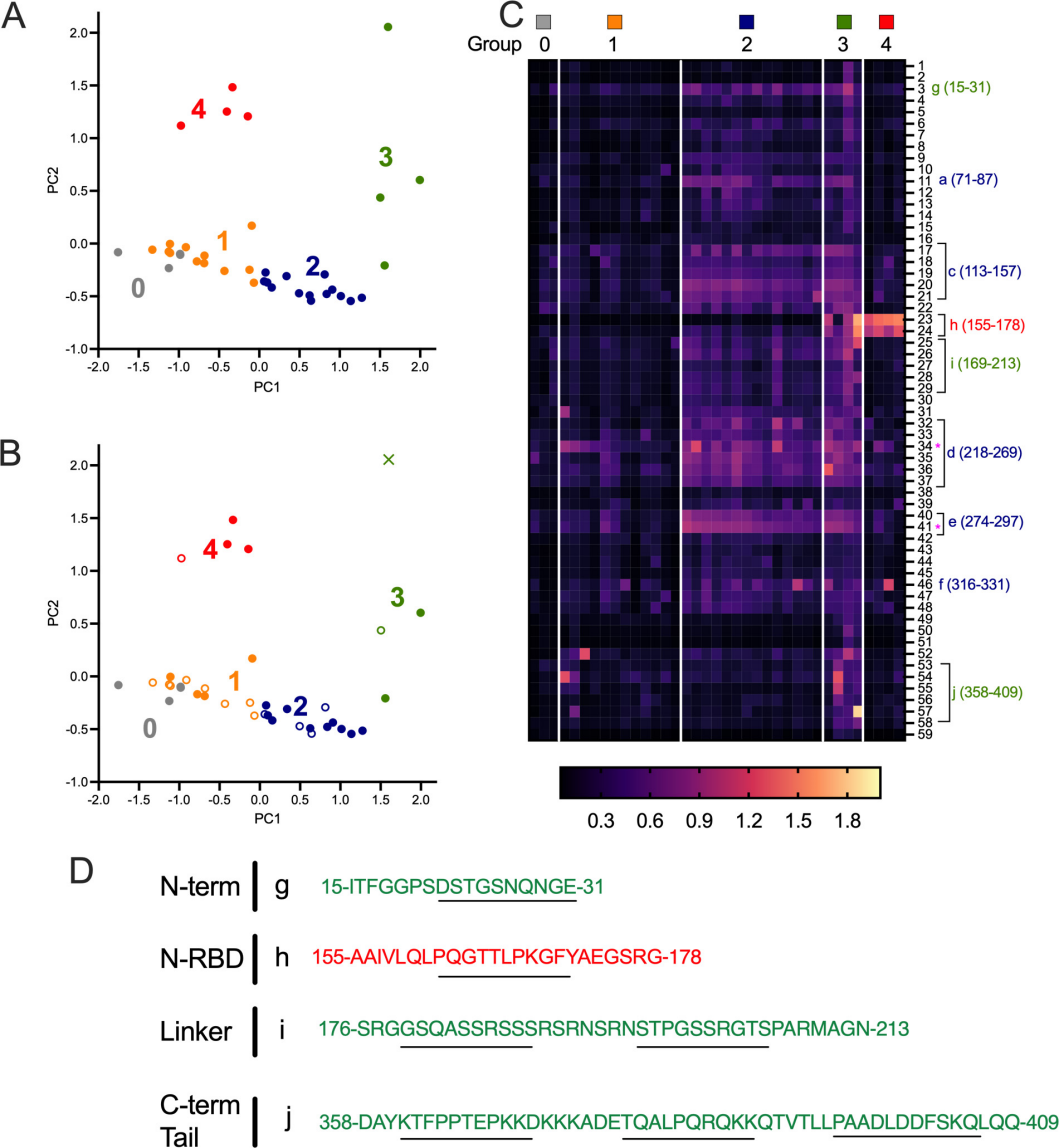
Private epitope identification by *k*-means clustering. (A) PCA representation of antibody profiles based on raw ELISA abundance data with *k*-means cluster labels overlaid. Group 0 represents control sera. (B) PCA representation of ELISA data with *k*-means clustering depicting patients treated in the ICU (solid circles) versus those not treated in the ICU (hollow circles). One patient death is designated with an X in group 3. (C) Magenta heatmap of raw optical read data with peptides in rows and each column representing a single patient. The most prevalent public peptides are marked with magenta asterisk. Peptide regions of interest are indicated at the right as detailed in Fig. 1B and panel D. (D) Sequences of antigenic peptides are listed with newly identified peptides resulting from group 3 in green and from group 4 in red. Overlapping 10- or 11-mer sequences are underlined. Regions g to j are labeled in order of sequence of N.

First, we performed a PCA on the peptide profile from each serum sample to reduce noise before clustering (Fig. 3A). A *k*-means clustering algorithm was used on the top two principal components to separate the 34 participants (and controls) into 4 groups that represent 4 distinct antibody response profiles (Fig. 3A). The optimal number of clusters was determined using the elbow method. Participant outcomes (hospitalized without ICU admission [non-ICU], hospitalized with ICU admission [ICU], and death) were overlaid (Fig. 3B), and antibody response by cluster was visualized as a heatmap (Fig. 3C). The mean responses across the peptides for each cluster are also shown as a line plot (Fig. S1).

Group 1 (Fig. 3A, orange, *n *= 12) displayed low antibody titers against nearly all peptides within the N protein (Fig. 3C; Fig. S1) and clustered tightly with the negative-control samples (Fig. 3A, group 0, gray). This group also contained the highest proportion of non-ICU participants (Fig. 3B, *n *= 8/12).

Group 2 (Fig. 3A, blue, *n *= 14) was the largest group and had antibody responses that were dominated by public epitopes identified in our prior analysis (Fig. 2, Fig. 3C, and Fig. S1). This group also contained the highest proportion of ICU participants (Fig. 3B, *n *= 10/14).

Group 3 (Fig. 3A, green, *n *= 4) followed a similar serodominant antibody response pattern to group 2, but with higher average absorbance values and unique responses to a few additional peptides (Fig. 3C; Fig. S1). This included a higher overall response to peptide 3 (aa 15 to 31, designated region g) in the N-terminal domain and to peptides 23 and 24 (aa 155 to 178, designated region h) corresponding to the N-RBD. Group 3 also showed a strong response to the linker region covered by peptides 25 to 26 and 28 to 29 (aa 169 to 213, designated region i), with the highest overall response for peptides 26 (aa 176 to 192) and 28 (aa 190 to 296). In addition, group 3 patients had uniquely robust antibody responses to several C-terminal tail peptides 52 to 58 (aa 358 to 409, designated region j). Overall, group 3 patients, in addition to the immunodominant profile captured in group 2, reacted more broadly to peptides of the N protein specifically in the unstructured regions.

Group 4 (Fig. 3A, red, *n *= 4) had the most unique response profile, with low reactivity to nearly all peptides, with the exception of a very strong response to peptides 23 and 24 (region h, aa 162 to 171), which share the overlapping sequence PQGTTLPKGF (Fig. 3C and D). Group 4, and to a lesser extent, group 3, was characterized by the dominance of the response to region h in the N-RBD, as most patients who were represented in groups 1 and 2 showed no response to this epitope (Fig. 3C; Fig. S1). These responses seemed to occur in participants who experienced more severe outcomes (2 non-ICU, 5 ICU, 1 death between groups 3 and 4), though more participants would be needed to assess significance (Fig. 3B).

### Antigenic peptides are distinct from RNA binding regions of the N-RBD and N-DD.

**FIG 4:**
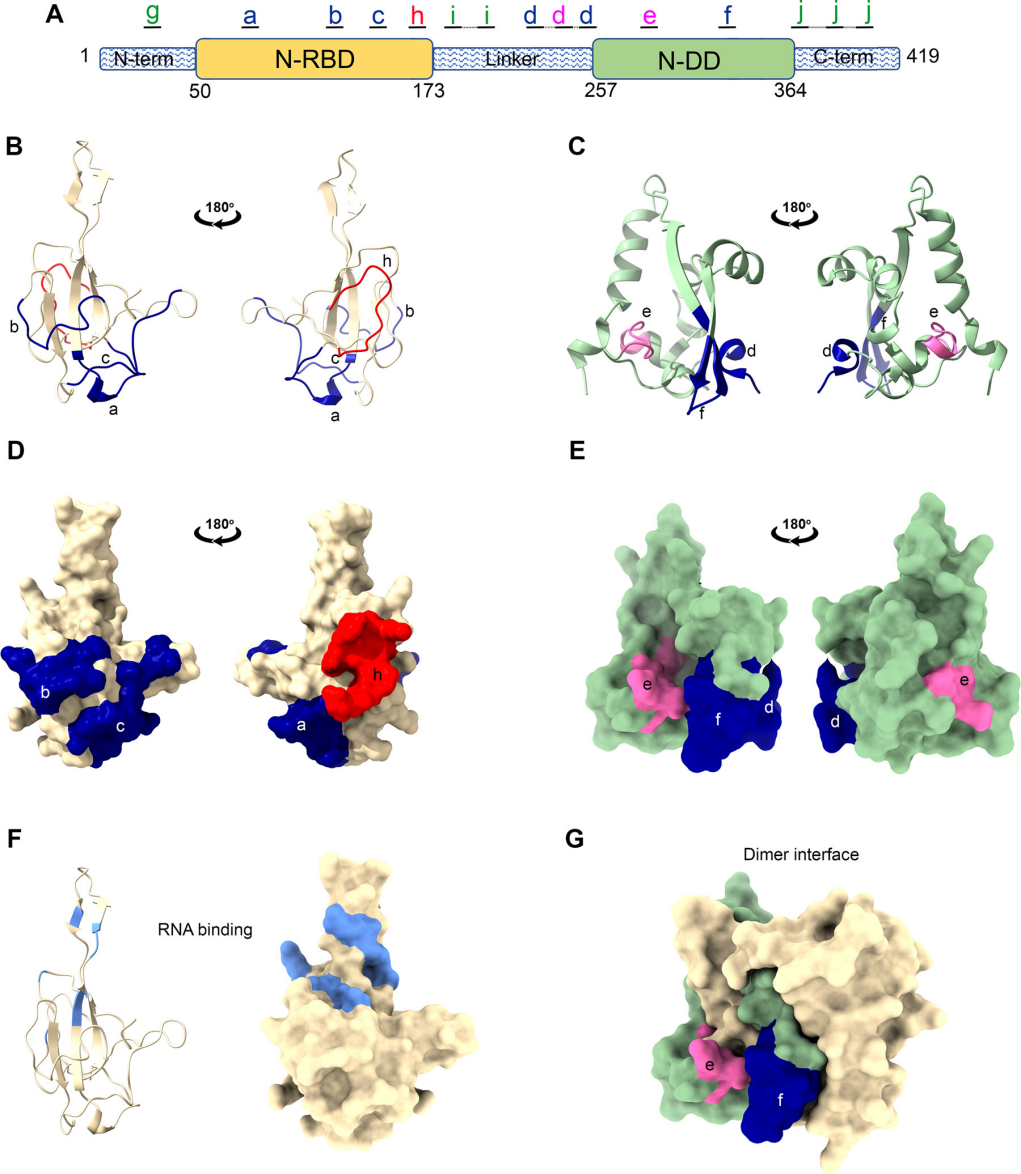
Mapping of antigenic peptides to structures of N-RBD and N-DD. Locations of peptides are shown on the schematic diagram (A) and ribbon (B and C) and surface (D and E) representations of the structure of the N-RBD (B and D, PDB accession no. 6VYO) and N-DD (E and F, PDB accession no. 6WJI). Colored regions are labeled as in Fig. 2B and Fig. 3D, with peptide regions that generated modest responses shown in blue (peptide regions a, b, c, d, and f), high responses shown in pink (region e), and the highly reactive private epitope seen in group 4 shown in red (region h). (F) The residues shown by nuclear magnetic resonance to bind RNA are highlighted in light blue in the ribbon. Surface projection diagrams of the N-RBD in the same orientation as shown at the left of panels A and C. (F) The surface projection of the dimer of N-DD is shown with both chains A (green) and B (tan). Chain A is in the same orientation as the left of panel E.

To structurally contextualize the epitopes of the N protein, we mapped the immunogenic peptides to the crystal structures (Fig. 4). Four of the antigenic regions were derived from the disordered, flexible regions of the protein (Fig. 4A), including the N-terminal arm (region g, aa 15 to 31), the linker region (regions i and d, aa 169 to 213 and 218 to 269, respectively), and the C-terminal tail (region j, aa 358 to 409). The peptide from aa 235 to 245 (region d) was particularly immunogenic, eliciting the strongest response in more than 50% of patients. Unfortunately, no structures are yet available for these flexible regions.

Six of the immunogenic peptides (regions a, b, c, h, e, and f), as well as part of region d, mapped to either the N-RBD or N-DD (Fig. 4A), consistent with our prior finding that both stable domains are antigenic (34). Visualization of these peptide positions on the crystal structures revealed that most of these mapped to flexible loops in the N-RBD and N-DD (Fig. 4B and C), and they were all solvent exposed (Fig. 4D and E). Furthermore, the peptides mapped to regions of the N-RBD distinct from the RNA binding regions, which were identified previously by nuclear magnetic resonance (35) (Fig. 4F). In the N-DD, the immunogenic peptides mapped to regions that projected away from the dimer interface and thus are exposed even in the dimerized form (Fig. 4G).

Altogether, these data and analyses indicate that the most immunogenic peptides map to either the flexible linker regions of the N protein or map to regions within the N-RBD and N-DD domains that are freely accessible and not bound by RNA or buried by dimerization.

### Amino acid changes in common SARS-CoV-2 variants occur in N antigenic regions.

**FIG 5:**
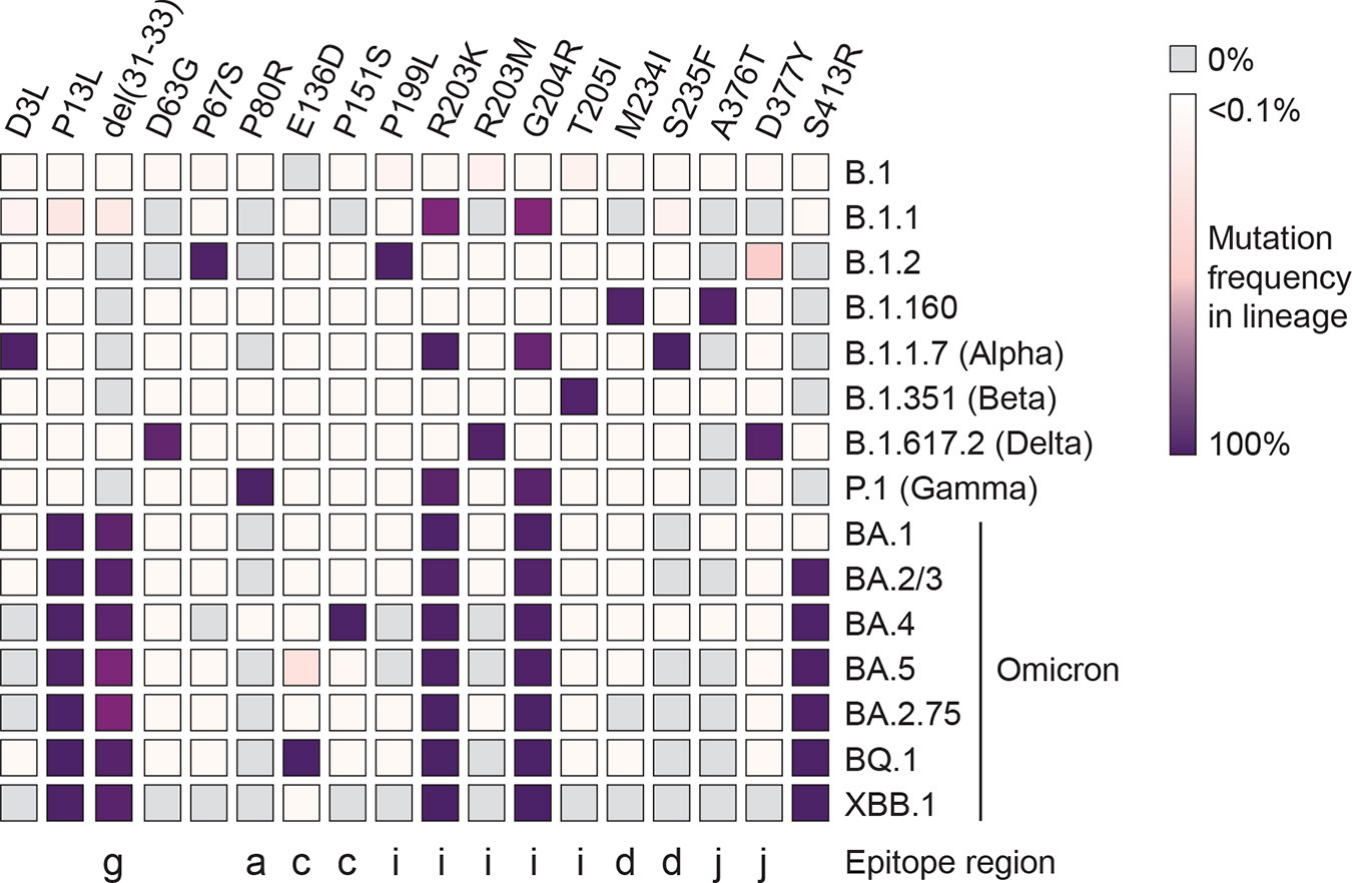
Mutations in N protein across major SARS-CoV-2 lineages. Mutational frequency in the N protein across 15 SARS-CoV-2 lineages is depicted as a heatmap. Only mutations that achieve at least 75% prevalence in at least one of the above-described Pango lineages are included.

The ongoing evolution of SARS-CoV-2 has resulted in the periodic emergence of variants with enhanced fitness that have spread broadly throughout the population. Early SARS-CoV-2 lineages were defined by only a few mutations relative to the reference viral genome reported from Wuhan, China (36). While most early lineages had no mutations in the N protein, lineage B.1.1 arose with two adjacent mutations in the linker region, R203K and G204R (Fig. 5), which have been associated with enhanced infectivity and viral fitness (37). Late in 2020, regionally specific lineages carrying a couple of additional N protein mutations became predominant, including B.1.2 (P67S and P199L) in North America and B.1.160 (M234I and A376T) in Europe. These lineages would later be displaced by variants of concern (VOCs) that arose independently in different parts of the globe, including the Alpha variant in the United Kingdom (with N mutations D3L, R203K, G204R, and S235F), the Beta variant in South Africa (with N mutation T205I), and the Gamma variant in Brazil (with N mutations P80R, R203K, and G204R). The Delta variant, which was first reported in India, would become predominant worldwide by the end of 2021, most sublineages of which carried three N mutations (D63G, R203M, and D377Y). The R203M mutation was found to drastically enhance viral infectivity, similar to the R203K and G204R mutations found in other VOCs (38).

In late 2021, the emergence of the Omicron variant led to the rapid displacement of all former lineages. The Omicron VOCs all have N mutation P13L, deletion of amino acids 31 to 33, R203K, and G204R. The Omicron variant has diversified substantially since its emergence into additional sublineages, most of which carried an additional N protein mutation S413R (BA.2, BA.4, BA.5, etc.). Sublineages of the Omicron variant have been predominant worldwide since then and currently represent all circulating VOCs, with some sublineages carrying additional N mutations (i.e., P151S in BA.4 and E136D in BQ.1) (Fig. 5).

The emergence of N mutations can impact antibody responses and could impact antigen-based monitoring. Notably, many of the lineage-defining mutations in the N protein map within one of the previously described, highly prevalent public epitopes (P80R in region a, E136D and P151S in region c, and M234I and S235F in region d), with others mapping to one of the patient-specific private epitopes (31 to 33 deletion in region g; P199L, R203K/M, G204R, and T205I in region i; and A376T and D377Y in region j) (Fig. 5). While R203K/M and G204R have been previously linked to enhanced infectivity and viral fitness (37, 38), it remains unknown if these or other mutations mapping to N epitopes result in altered immunogenicity or immune escape.

## DISCUSSION

Numerous studies have established that patients with COVID-19 infection develop serum responses to the SARS-CoV-2 N protein (3, 4, 24, 39), and we previously showed that these antibodies can develop to both the full-length N protein and the structured regions, N-RBD and N-DD (34). Diagnostic tests that depend on identification of antibodies to the N protein have been widely deployed to detect evidence of prior SARS-CoV-2 infection. Unlike antibody responses to the S protein, which is included in most SARS-CoV-2 vaccines, N protein responses can more readily discriminate individuals with prior infection from those previously vaccinated. For the purposes of vaccine development and for understanding potential changes in antigenicity in SARS-CoV-2 variants or cross-reactivity with other coronaviruses, there is ongoing interest in identifying strong immunogenic epitopes within the N protein using smaller peptide arrays.

In our study, we used a library of overlapping 17-mer peptides (see Table S2 in the supplemental material) to map linear N protein epitopes with 10-aa resolution (Table 2) and analyzed the data to identify both public epitopes that induced an antibody response in most participants (Fig. 2) and private epitopes that resulted in high reactivity, but in only a subpopulation of patients (Fig. 3). This study revealed that the most prevalent highly reactive peptides were located toward the end of the linker domain (aa 235 to 245, region d) and within the N-DD (aa 281 to 290, region e). Region d and three other immunogenic regions (g, i, and j) identified in this study map to the unstructured regions of the N protein, including the N-terminal, linker, and C-terminal tail regions, respectively.

**TABLE 2:** Comparison of antigenic N peptides identified in this study with prior studies

Peptide no.	Amino acids	Sequence (N→C)	Region	Domain	Group(s)	Data for peptides identified in other studies	
						No.	Reference(s)
3	22–31	DSTGSNQNGE	g	NTD	2, 3	4	23 – 25
11	74–84	INTNSSPDDQI	a	N-RBD	2, 3	2	23, 25
17	117–126	PEAGLPYGAN	b	N-RBD	2, 3	1	25
20, 21	141–150	TPKDHIGTRN	c	N-RBD	2, 3	2	25, 27
23, 24	162–171	PQGTTLPKGF	h	N-RBD	3, 4	7	24–26, 28–30, 32
25, 26	169–178	KGFYAEGSRG	i	Linker	3	6	23–26, 32
28, 29	197–206	STPGSSRGTS	i	Linker	3	4	23–25, 31
32	221–231	LLLLDRLNQLE	d	Linker	2, 3	5	24, 27–29, 31
34	235–245	SGKGQQQQGQT	d	Linker	1, 2, 3, 4	3	23 – 25
36	249–259	KSAAEASKKPR	d	Linker	2, 3	5	23–25, 27, 29
40, 41	281–290	QTQGNFGDQE	e	N-DD	2, 3	1	25
46	323–332	EVTPSGTWLT	f	N-DD	2, 3, 4	1	25
52	361–371	KTFPPTEPKKD	j	C-term tail	3	9	23–30, 32
54, 55	379–388	TQALPQRQKK	j	C-term tail	3	8	24–30, 32
57	396–406	PAADLDDFSKQ	j	C-term tail	3	6	24, 26–29, 32

In addition to epitopes in the flexible interdomain regions, we also identified epitopes mapping within the unstructured surface-exposed loops of the stable folded N-RBD (Fig. 4B, D, and F). The most reactive peptide in N-RBD was located at aa 162 to 171 (region h), although antibodies against this peptide were identified in only a small fraction of patients (groups 3 and 4). Unique to our study, we identified two broadly reactive epitopes within the N-DD, a highly reactive peptide at aa 281 to 290 (region e) and a moderately reactive peptide at aa 323 to 332 (region f). Immunogenic epitopes have not been previously experimentally mapped to this region. We found that these epitopes are also surface exposed in regions that are not masked by the dimer interface. The identification of immunogenic epitopes in this domain is consistent with our prior published studies that antibody responses occur against both the N-RBD and the N-DD (34).

To place our results into context, we conducted a literature review examining studies that either predicted or experimentally determined antibody responses to the SARS-CoV-2 N protein (Table 2). Several previous publications have used immunoinformatic approaches to predict epitopes (23–26). Early in the pandemic, Grifoni et al. (26) used a computational approach and predicted aa 43 to 65, 154 to 175, and 356 to 404 as potential B cell epitopes. Two of these regions were identified by our study as private epitopes present in a subset of our cohort. Amino acids 154 to 175 corresponded to region h, the highly reactive peptide from group 4, and amino acids 356 to 404 corresponded to the C-terminal tail region we designated j, a region associated with strong responses in group 3. Additionally, we noted antibody responses to five of the six N protein epitopes predicted by Oliveira et al. (23) as probable B-cell epitopes, including aa 21 to 32 (region g), 76 to 82 (region a), 176 to 206 (region i), 235 to 243, 249 to 263 (region d), and 363 to 379 (region j). Overall, our data most closely mirrored the epitope predictions of Crooke et al. (25). Of the 14 potential epitopes identified by computational approaches, we experimentally identified nine of them. Notably, Crooke et al. (25) was the only study of those reviewed that predicted responses to our two unique reactive epitopes within the N-DD, aa 281 to 290 (region e) and 323 to 332 (region f).

Shrock et al. (24) deployed a phage display platform (VirScan) that encoded 20-mer peptides tiled every five amino acids across the entire SARS-CoV-2 proteome to profile antibody responses. Their study revealed that most patients seroconvert and produce antibodies against the N protein and that responses to the N protein were stronger in hospitalized patients. Using the 20-mer library, four highly reactive epitopes were identified that were recurrently targeted by antibodies in at least 15% of patients, aa 222 to 242, 240 to 260, 365 to 385, and 386 to 406, which corresponded to public epitopes from our study in region d and private epitopes in region j. All of the highly reactive peptides map to the unstructured linker or C-terminal tail regions. Finally, Shrock et al. (24) also identified stronger antibody responses in hospitalized patients against amino acids 161 to 181. These responses corresponded to regions h and i in our study, which represent surface-exposed regions of the N-RBD and the beginning of the unstructured linker domain.

Studies that have evaluated proteome or linear peptide overlapping arrays are most similar to the study we conducted and have yielded the most granular insights into N protein antibody responses (27 to 32). Amrun et al. (30) scanned an 18-mer peptide library, identified aa 153 to 170 as reactive against pooled plasma from 18 patients, and found that a strong response to this peptide correlated with more severe disease. This peptide overlaps with our region h, identified as a private epitope in groups 3 and 4. These data are consistent with our finding that 5 of 8 patients in these groups were treated in the ICU, and group 3 contained the only patient death in our cohort. In a smaller cohort of patients (*n *= 10), Wang et al. used a linear epitope array of 15-mer peptides with a five-residue overlap to define antibody responses to the N protein (29). This study found eight distinct peptides which elicited strong IgG antibody responses, including three (aa 166 to 170, 226 to 230, and 366 to 400) which overlapped with regions h, d, and j in our study. Using an identical peptide-overlapping strategy in a larger cohort (*n *= 89 patients) (29), Voss et al. found a total of 26 N protein peptides that elicited an antibody response (27). The strongest response was to eight peptides that mapped to the C-terminal tail (aa 355 to 400), with the most robust responses to aa 361 to 375 and aa 381 to 395, consistent with our highly reactive private group 3 epitopes in the C-terminal tail region j. Epitopes with medium response intensity were identified for nine peptides mapping to the linker domain, with aa 221 to 235 and 246 to 260 showing the strongest responses. These peptides match our study’s most highly reactive public epitope in region d. The final epitope with a strong response was aa 156 to 170 within the N-RBD, corresponding to region h. In contrast to the study by Amrun et al. (30) and our data, a strong reaction to this peptide correlated with less severe disease in this cohort. In a similar study, Holenya et al. (28) mapped high-resolution epitopes from 24 patients using a 15-mer peptide array with an 11-amino-acid overlap. They, too, identified similar immunoreactive epitopes at aa 161 to 171 (region h), 221 to 231 (region d), and three peptides within the C-terminal tail domain (region j). Finally, Hotop et al. (31) used a 15-mer overlapping peptide microarray to investigate responses in 36 SARS-CoV-2 patients. They noted that antibody responses to aa 220 to 234 (region d) within the linker domain were associated with more severe disease. Region d was one of the two most prevalent immunodominant regions in our study, with brisk antibody responses within groups 2, 3, and 4, though our cohort was too small to draw clear conclusions on the association between antibody response and patient outcomes. Camerini et al. (32) took a slightly different approach and investigated serum responses to the N protein using the full-length N protein as well as larger 100-, 50-, and 30-amino-acid peptide fragments. Significant responses occurred in aa 301 to 400 that were resolved to the last 69 amino acids (350 to 419). This result parallels our findings that the C-terminal tail (region j) has strong epitopes and that responses to this region are variable across patient samples. Unlike our peptide array, Camerini et al. detected no responses to peptide fragments smaller than 30 amino acids.

Ultimately, the antigenicity of linear peptides mapping to the central linker region of the N protein, including peptide d, was consistent with nearly all other epitope-mapping studies (27–32). In total, these data support our finding that region d is a recurring highly prevalent epitope from the linker region that was identified in five other studies. The broad computational and *in vitro* support of serological responses to region d suggest that SARS-CoV-2 diagnostic test accuracy could be improved if diagnostics included peptides within this region. The recurring identification of region h in the N-RBD in nine other studies and j in the C-terminal tail in eight other studies confirms these are strong epitopes (Table 2), although all studies indicate these are strong epitopes in only a fraction of patients (private epitopes). Similar to region d, including peptides within regions h and j may improve diagnostic test accuracy.

The most significant difference compared to other studies is our identification of highly reactive peptides in the N-DD. We identified a predominant public epitope in region e that led to strong antibody responses in both groups 2 and 3 and region f that led to positive responses in more than 50% of all patients and high responses in a portion of patients in groups 2, 3, and 4. No other experimental studies identified epitopes in the N-DD, and only one computational study predicted epitopes in this stable folded domain (25). The nature of this discrepancy remains unclear, especially given the paucity of N mutations that map to this region (Fig. 5). Determination of the role of antibody responses to N-DD regions e and f and their impact on disease outcome would benefit from sampling larger, longitudinal patient cohorts.

The limitations of our study are the small cohort size, delay between sample acquisition and analysis, and the exclusive use of linear peptide fragments. Despite these limitations, we defined the dominant antibody response profile of our cohort and clustered patients into four unique groups based on distinct antibody response profiles. Given the small size of our cohort, we were not able to correlate specific antibody responses with patient outcome. However, most of the published studies likewise examined small patient cohorts, such that our study revealed the benefit of an aggregate analysis confirming the identification of dominant epitopes and suggested that region h private peptides are correlated with poor patient outcomes. Another limitation of our study is the long lag between data collection and analysis, as in the interval, our structural biology group focused on other COVID priorities, including drug development. This has not impacted our results, as a literature review demonstrated that our data are highly consistent with other published studies, and our analysis benefited from comparison to the larger aggregate sample size from 10 studies. Given the constant, albeit slow, evolution of the SARS-CoV-2 virus, an ideal data set would compare serological responses longitudinally over the course of the pandemic to assess for changing responses to the N protein. Inclusion of patients with a wider range of clinical symptoms and with and without history of prior vaccination would also provide important comparisons. Last, our study focused exclusively on linear peptide fragments to assess antibody response. This type of analysis excludes the possibility that confirmational epitopes are important for immunogenicity. However, in a prior study, we previously showed that the level of the antibody response to full-length N-protein, N-RBD, and N-DD also did not correlate with patient outcome (34).

Ultimately, our study adds to the growing body of literature on antibody responses to the SARS-CoV-2 N protein and is unique in that it places those responses into the context of the structure of the protein. Such analyses are critical for efforts to produce subunit vaccines as well as improved sensitivity and specificity of newly developed diagnostics, which may use peptide-specific antibodies rather than full-length N protein as the basis for seroconversion. Clearly, the SARS-CoV-2 N protein is highly and broadly immunogenic, and antibody responses are robust and durable despite the duration of the current pandemic. Though much attention has been devoted to the SARS-CoV-2 spike protein, studies such as ours suggest that broadening the evaluation of patient antibody responses to include the N protein will undoubtedly inform diagnostic development and inform treatment approaches. Although short of the penultimate goal of developing accurate tools to predict patient outcomes, studies that combine structural biology with peptide-tiled arrays such as ours continue to be powerful tools to study and compare antibody responses to evolving variants of concern of SARS-CoV-2.

## MATERIALS AND METHODS

### Protein expression, purification, and structure determination.

Methods for expression and purification of the N-DD (amino acids 247 to 364, previously referred to as the N-CTD) as recombinant protein in Escherichia coli were previously described (34). The pure N-DD protein was set up as 2-μL crystallization sitting drops (1 μL protein/1 μL reservoir solution) in 96-well plates and equilibrated with commercially available Classics II, JCSG+, MPD, and ComPAS suites. Diffraction-quality crystals appeared in 6 days from condition D8 of the Classics II screen (0.1 M HEPES, pH 7.5, and 25% [wt/vol] polyethylene glycol 3350 [PEG 3350]). Diffraction data were collected at the 21-ID-F Life Science Collaborative Access Team (LS-CAT) at the Advanced Photon Source, Argonne National Laboratory. The data set was processed and scaled with the HKL-3000 suite (40). The structure was solved by molecular replacement with Phaser (41) from the CCP4 suite (42) using the crystal structure of the oligomerization domain from SARS coronavirus TW1 (PDB accession no. 2CJR) (43) as a search model. The initial solution went through several rounds of refinement in REFMAC v5.8.0258 (44) and manual model corrections using Coot (45). The water molecules were generated using ARP/wARP (46), and chloride ions were added to the model manually during visual inspection in Coot. Translation-Libration-Screw (TLS) groups were created by the TLSMD server (47) (http://skuld.bmsc.washington.edu/~tlsmd/), and TLS corrections were applied during the final stages of refinement. Molprobity (48) (http://molprobity.biochem.duke.edu/) was used for monitoring the quality of the model during refinement and for the final validation of the structure.

The final models and diffraction data were deposited to the Protein Data Bank (https://www.rcsb.org/) with the assigned PDB accession no. 6WJI. The plasmid used for protein production of N-DD is available at BEI Resources (catalog no. NR-52434).

### Serum collection and processing from COVID-19 patients.

Individuals over the age of 18 admitted to Northwestern Memorial Hospital with a positive PCR-based COVID-19 diagnostic test, who provided informed consent themselves or through an appropriate surrogate, were enrolled in the study per institutional review board (IRB) approval no. STU00206850. Methods and procedures for specimen collection, processing, and deposition to the Northwestern COVID-19 BioBank have been previously described (34). For this study, only the first available serum specimen from 34 participants was used. All available clinical data from the Northwestern Medicine Enterprise Data Warehouse (NMEDW) and from electronic chart review were pulled for this subset of hospitalized participants per institutional review board approval no. STU00212267. These NMEDW data were utilized to determine demographics, clinical assessments, symptom onset, laboratory measurements, COVID-19 disease severity, and hospital outcomes (intensive care unit [ICU] care and death) for the study analyses.

### ELISA.

A 59-peptide array covering the amino acid sequence of the N protein as 17 or 13 mer with 10-amino-acid overlaps was obtained from BEI Resources (catalog no. NR-52404). One milligram of lyophilized peptide was resuspended in 1 mL phosphate-buffered saline (PBS) and diluted to 5 μg/mL in PBS, and 100 μL was added to each well of a 96-well microtiter plate (Nunc-Immuno MicroWell 96-well solid plates; Fisher Scientific). Plates were stored at 4°C for 24 to 48 h and washed three times with 250 μL PBS plus 0.05% Tween 20 (PBS-T). Plates were blocked with 200 μL PBS-T plus 2% bovine serum albumin for 4 h. Blocking buffer was removed and replaced with 100 μL patient sera diluted 1:1,000 in blocking buffer. Plates were incubated for 1 h at room temperature and then washed three times with PBS-T. One hundred microliters of 1 μg/mL horseradish peroxidase (HRP)-conjugated anti-human IgG in blocking buffer was added to wells and incubated for 1 h at room temperature with shaking. The plate was washed three times with 250 μL PBS-T. We added 100 μL 3,3′,5,5′-tetramethylbenzidene (TMB) substrate to wells for 1 min, and then reactions were stopped with 100 μL stop solution. Absorbance was read at 450 nm on a Molecular Devices SpectraMax M3 spectrophotometer using SoftMax Pro v6.5.1 software.

### Clustering analysis.

Absorbance values were first normalized to the control and then subjected to a principal-component analysis (PCA). *k*-Means is an algorithm that seeks to partition samples into clusters using Euclidean distance. We performed *k*-means in the PCA feature space to identify distinct clusters of patients with similar absorbance profiles. The number of optimal clusters was determined via the elbow method, i.e., a method that visually identifies a point at which additional clusters no longer reduce the within-cluster distances. All analyses were performed in Python (v3.8.8). PCA and *k*-means were run using PCA and KMeans modules from the sklearn package (v0.24.1).

### N protein mutational analysis.

The frequency of mutations in the N protein across 15 prominent SARS-CoV-2 lineages was calculated using available whole-genome sequences from the Global Initiative on Sharing Avian Influenza Data (GISAID) database at https://nextstrain.org/, accessed 2 December 2022. Only mutations that reached at least 75% frequency in at least one of the examined lineages were included in the analysis.

### Data availability.

The structure of N protein N-DD was deposited to the Protein Data Bank (https://www.rcsb.org/) with accession no. 6WJI. The plasmid for expression of the N-DD (catalog no. NR-52434) and the peptide library used in this study (catalog no. NR-52404) are available from BEI Resources (www.beiresources.org).
