# Supplementary material for: Serodominant SARS-CoV-2 Nucleocapsid Peptides Map to Unstructured Protein Regions
Source: Microbiol Spectr. 2023 May 16;11(3):e00324-23. doi: 10.1128/spectrum.00324-23 (PMC10269789; doi:10.1128/spectrum.00324-23)
Supplement: Supplemental file 1 — Tables S1 and S2 and Fig. S1. Download spectrum.00324-23-s0001.pdf, PDF file, 0.08 MB [file spectrum.00324-23-s0001.pdf]

Supplemental Table 1. N-DD structure determination data refinement

**Data processing**	**PDB ID 6wji**
Structure	N-DD
Beamline	APS 21-ID-F
Wavelength (Å)	0.9787
Resolution range (Å)	30.00-2.05 (2.07-2.05)
Space group	$P2_12_12_1$
Cell parameters (Å, °)	*a*=43.61, *b*=122.32, *c*=130.63; α=90.00; β=90.00; γ=90.00
Unique reflections	43,336 (1,770)
Multiplicity	6.2 (5.5)
Completeness (%)	97.0 (80.6)
Mean I/sigma(I)	9.7 (2.6)
Wilson B-factor ($Å^2$)	17.1
R-merge[a]	0.171 (0.630)
CC1/2[b]	0.976 (0.698)
**Refinement**	
Resolution range (Å)	29.88-2.05
Reflections work/test	43,303/2,192
R_{work}/R_{free}[c]	0.187/0.228
Number of atoms	5,800
macromolecules	5,196
Ligands (Cl^-)/solvent (H_2O)	9/622
RMSD (bonds) (Å)	0.004

RMSD (angles) (°)	1.493
Ramachandran favored (%)	98.00
Ramachandran allowed (%)	2.00
Ramachandran outliers (%)	0.0
Rotamer outliers (%)	1.30
Clashscore	4.0
Average B-factor (Å^2)	20.1
macromolecules	19.4
ligands (Cl^-)	29.6
solvent (H_2O)	25.9

Supplemental Table 2. N protein amino acid sequences within peptide array

Peptide	Length	Sequence	a.a.	Domain
1	17	MSDNGPQNQRNAPRITF	1-17	NTD
2	17	NQRNAPRITFGGPSDST	8-24	NTD
3	17	ITFGGPSDSTGSNQNGE	15-31	NTD
4	17	DSTGSNQNGERSGARSK	22-38	NTD
5	17	NGERSGARSKQRRPQGL	29-46	NTD
6	17	RSKQRRPQGLPNNTASW	36-52	N-RBD
7	17	QGLPNNTASWFTALTQH	43-59	N-RBD
8	17	ASWFTALTQHGKEDLKF	50-66	N-RBD
9	17	TQHGKEDLKFPRGQGVP	57-73	N-RBD
10	17	LKFPRGQGVPINTNSSP	64-80	N-RBD
11	17	GVPINTNSSPDDQIGYY	71-87	N-RBD
12	17	SSPDDQIGYYRRATRRI	78-94	N-RBD
13	17	GYYRRATRRIRGGDGKM	85-101	N-RBD
14	17	RRIRGGDGKMKDLSPRW	92-108	N-RBD
15	17	GKMKDLSPRWYFYYLGT	99-115	N-RBD
16	17	PRWYFYYLGTGPEAGLP	106-122	N-RBD
17	17	LGTGPEAGLPYGANKDG	113-129	N-RBD
18	17	GLPYGANKDGIIWVATE	120-136	N-RBD
19	17	KDGIIWVATEGALNTPK	127-143	N-RBD
20	17	ATEGALNTPKDHIGTRN	134-150	N-RBD
21	17	TPKDHIGTRNPANNAAI	141-157	N-RBD
22	17	TRNPANNAAIVLQLPQG	148-164	N-RBD
23	17	AAIVLQLPQGTTLPKGF	155-171	N-RBD
24	17	PQGTTLPKGFYAEGSRG	162-178	N-RBD, Linker
25	17	KGFYAEGSRGGSQASSR	169-185	N-RBD, Linker
26	17	SRGGSQASSRSSSRSRN	176-192	Linker
27	17	SSRSSSRSRNSSRNSTP	183-199	Linker
28	17	SRNSSRNSTPGSSRGTS	190-206	Linker
29	17	STPGSSRGTSPARMAGN	197-213	Linker
30	17	GTSPARMAGNGGDAALA	204-220	Linker
31	17	AGNGGDAALALLLLDRL	211-227	Linker
32	17	ALALLLLDRLNQLESKM	218-234	Linker
33	17	DRLNQLESKMSGKGQQQ	225-241	Linker
34	17	SKMSGKGQQQQGQTVTK	232-248	Linker
35	17	QQQQGQTVTKKSAAEAS	239-255	Linker, N-DD
36	17	VTKKSAAEASKKPRQKR	246-262	N-DD
37	17	EASKKPRQKRTATKAYN	253-269	N-DD

38	17	QKRTATKAYNVTQAFGR	260-276	N-DD
39	17	AYNVTQAFGRRGPEQTQ	267-283	N-DD
40	17	FGRRGPEQTQGNFGDQE	274-290	N-DD
41	17	QTQGNFGDQELIRQGTD	281-297	N-DD
42	17	DQELIRQGTDYKHWPQI	288-304	N-DD
43	17	GTDYKHWPQIAQFAPSA	295-311	N-DD
44	17	PQIAQFAPSASAFFGMS	302-318	N-DD
45	17	PSASAFFGMSRIGMEVT	309-325	N-DD
46	17	GMSRIGMEVTPSGTWLT	316-332	N-DD
47	17	EVTPSGTWLTYTGAIKL	323-339	N-DD
48	17	WLTYTGAIKLDDKDPNF	330-346	N-DD
49	17	IKLDDKDPNFKDQVILL	337-353	N-DD
50	17	PNFKDQVILLNKHIDAY	344-360	N-DD
51	17	ILLNKHIDAYKTFPPTE	351-367	N-DD
52	17	DAYKTFPPTEPKKDKKK	358-374	N-DD, CTD
53	17	PTEPKKDKKKKADETQA	365-381	CTD
54	17	KKKKADETQALPQRQKK	372-388	CTD
55	17	TQALPQRQKKQQTVTLL	379-395	CTD
56	17	QKKQQTVTLLPAADLDD	386-402	CTD
57	17	TLLPAADLDDFSKQLQQ	393-409	CTD
58	17	LDDFSKQLQQSMSSADS	400-416	CTD
59	13	LQQSMSSADSTQA	407-419	CTD

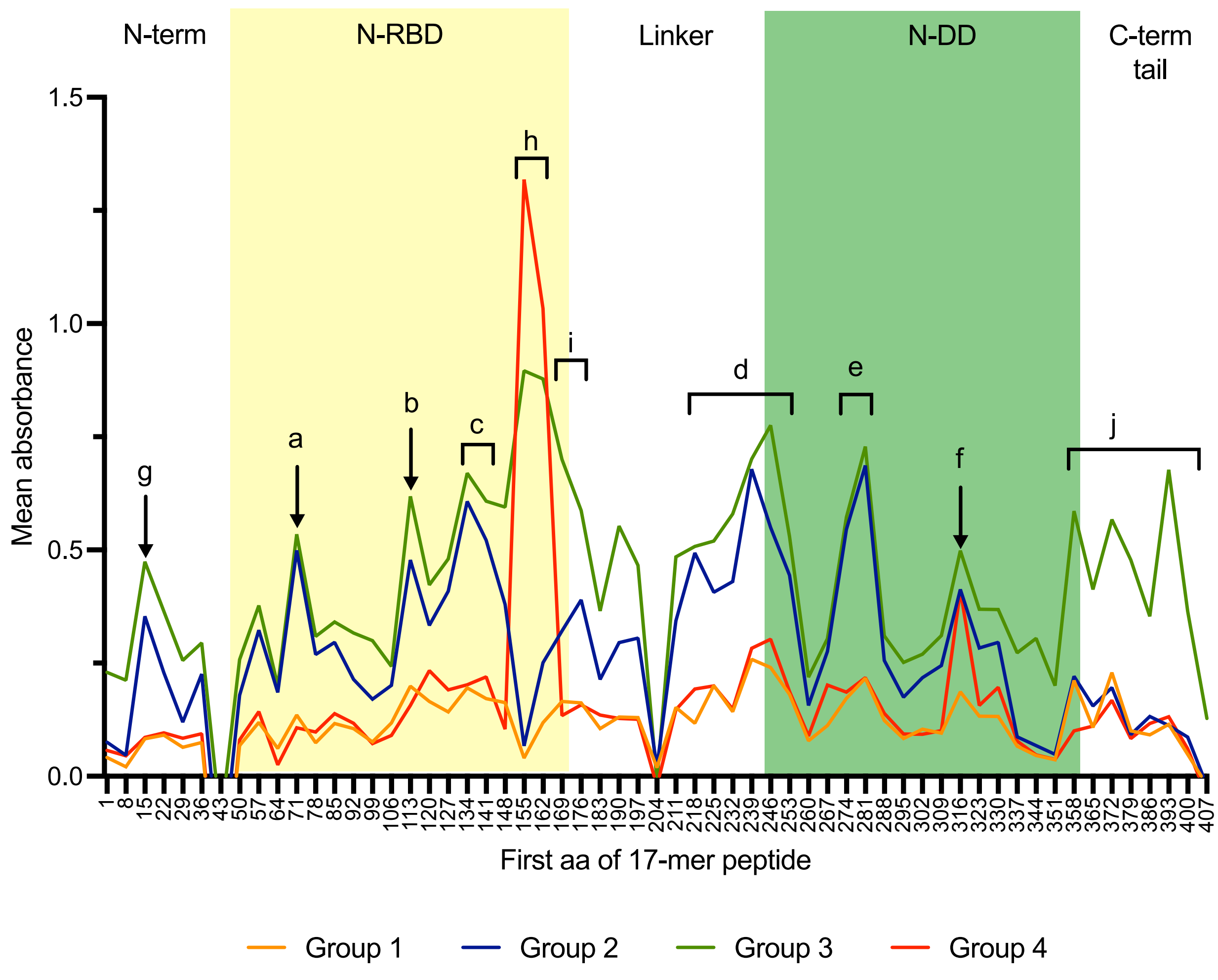

Suppl. Fig. 1. Epitope reactivity by peptide and cluster. The mean absorbance for each cluster group for each peptide. Regions **a-j** are indicated above the peaks.
